# Risk Tier, Variant Certainty, and Real-World Care Patterns in Breast Cancer Patients with Germline Alterations in Breast Cancer Susceptibility Genes

**DOI:** 10.3390/cancers18101499

**Published:** 2026-05-07

**Authors:** Tuba Baydaş, İlker Nihat Ökten, Filiz Özen, Süheyla Emre, İbrahim Çil, Metin Eser, Adnan Gündoğdu, Osman Cem Yılmaz, Mahmut Gümüş

**Affiliations:** 1 Department of Medical Oncology, Göztepe Prof. Dr. Süleyman Yalçın City Hospital, Istanbul 34722, Türkiye; ilkernihat@gmail.com (İ.N.Ö.); mgumus@superonline.com (M.G.); 2 Department of Medical Genetics, Göztepe Prof. Dr. Süleyman Yalçın City Hospital, Istanbul 34722, Türkiye; 3 Department of Medical Oncology, SBÜ Ümraniye Training and Research Hospital, Istanbul 34764, Türkiye; 4 Department of Medical Genetics, SBÜ Ümraniye Training and Research Hospital, Istanbul 34764, Türkiye; 5 Department of General Surgery, SBÜ Sancaktepe Şehit Prof. Dr. İlhan Varank Training and Research Hospital, Istanbul 34785, Türkiye; 6 Department of Breast Surgery, Istanbul Breast Center, Istanbul 34740, Türkiye

**Keywords:** breast cancer, germline testing, pathogenic variants, variants of uncertain significance, penetrance, hereditary cancer, molecular subtype

## Abstract

Background: The increasing use of multigene germline testing in breast cancer has expanded the detection of pathogenic/likely pathogenic (P/LP) variants and variants of uncertain significance (VUSs) across a broad range of breast cancer susceptibility genes. However, the extent to which pathogenic certainty and penetrance level are associated with distinct clinicopathological phenotypes and management patterns remains incompletely defined. Methods: In this multicenter retrospective study, we included 405 breast cancer patients with germline alterations in breast cancer susceptibility genes. Patients were classified into three groups: high-penetrance P/LP (*n* = 116), moderate/low-penetrance P/LP (*n* = 69), and VUS (*n* = 220). The primary endpoint was non-luminal invasive breast cancer phenotype, defined as HER2-positive or triple-negative disease versus luminal A/B disease. Multivariable logistic regression was performed to assess the association between genetic group and non-luminal phenotype, adjusted for age at diagnosis and family history. Secondary analyses evaluated management patterns across groups and examined a prespecified non-*BRCA* cohort. Results: Patients with high-penetrance P/LP variants were younger at diagnosis and had higher Ki-67 values than those in the other groups. Molecular subtype distribution differed significantly across groups, with triple-negative disease most frequent in the high-penetrance P/LP group (33.9%) compared with the moderate/low-penetrance P/LP (9.8%) and VUS (12.9%) groups. In multivariable analysis, high-penetrance P/LP status was independently associated with increased odds of non-luminal phenotype compared with VUS (OR 1.79, 95% CI 1.06–3.04; *p* = 0.029), whereas moderate/low-penetrance P/LP status was not (OR 0.96, 95% CI 0.48–1.91; *p* = 0.911). Management patterns also differed significantly. Initial mastectomy was performed in 65.4% of evaluable patients in the high-penetrance P/LP group, compared with 46.4% in the moderate/low-penetrance P/LP group and 45.5% in the VUS group. Final bilateral mastectomy was observed in 62.6%, 17.5%, and 9.9%, respectively (*p* < 0.001). In the prespecified non-*BRCA* analysis, non-*BRCA* P/LP status was not associated with non-luminal phenotype compared with non-*BRCA* VUS (OR 0.83, 95% CI 0.40–1.72; *p* = 0.609). Conclusions: Among breast cancer patients with germline alterations in breast cancer susceptibility genes, high-penetrance P/LP variants are associated with a distinct clinicopathological profile characterized by younger age at diagnosis, higher proliferative activity, and increased likelihood of non-luminal disease. In contrast, moderate/low-penetrance P/LP variants, especially VUSs, do not show the same degree of phenotype specificity. These findings support a framework in which pathogenic certainty and penetrance level remain central to the interpretation of multigene germline testing in breast cancer.

## Simple Summary

Multigene germline testing is now widely used in breast cancer, but the results it identifies are not equally informative. Some patients carry clearly pathogenic variants in high-risk breast cancer susceptibility genes, others carry pathogenic variants in genes with lower or less certain clinical impact, and many receive results classified as variants of uncertain significance. In this multicenter retrospective study, we examined how these different genetic result categories relate to tumor characteristics and real-world treatment patterns in 405 patients with breast cancer. We found that high-penetrance pathogenic variants were associated with younger age at diagnosis, higher tumor proliferative activity, and a greater likelihood of non-luminal disease, while lower-penetrance pathogenic variants and uncertain variants showed less distinct patterns. These findings support a more risk-tiered and clinically nuanced interpretation of germline test results in routine breast cancer care.

## 1. Introduction

Germline genetic testing has become an integral component of modern breast cancer care, with implications for systemic therapy selection, local treatment planning, risk assessment, and cascade testing for family members. Contemporary guidelines recommend broader access to germline testing for patients with breast cancer, particularly for *BRCA1/2*, while also supporting multigene panel testing when results are expected to inform personal or familial cancer risk [1,2,3,4,5]. As multigene hereditary cancer panels have become more widely used, alterations are increasingly identified across a broader spectrum of breast cancer susceptibility genes, including both high- and moderate-penetrance genes, along with a growing number of variants of uncertain significance (VUSs) [1,2,6,7].

The clinical implications of these findings are not uniform. Population-based and large-scale case–control studies have shown that the magnitude of breast cancer risk varies substantially by gene, with *BRCA1*, *BRCA2*, and *PALB2* generally conferring the highest risks, whereas genes such as *ATM* and *CHEK2* are more often associated with moderate penetrance [8,9,10,11]. These studies have also demonstrated that inherited pathogenic variants are linked to distinct tumor phenotypes. In particular, *BRCA1*, *BARD1*, *RAD51C*, and *RAD51D* are strongly enriched for triple-negative disease, whereas *ATM* and *CHEK2* are more commonly associated with hormone receptor-positive tumors. In addition, tumors arising in pathogenic-variant carriers are frequently of higher grade, further supporting biologic heterogeneity across hereditary breast cancer syndromes.

Despite these advances, important uncertainties remain in routine practice. Much of the literature has focused on *BRCA1/2*, whereas the interpretation of non-*BRCA* findings—particularly those involving moderate-penetrance genes—remains more complex. Reviews in this area have emphasized that the biologic and clinical implications of moderate-penetrance genes are less uniform than those of *BRCA1/2* and that additional real-world data are needed to refine counseling, surveillance, and risk-reduction strategies in this setting [10,12,13].

The challenge is even greater for VUSs, which are increasingly encountered as panel testing expands. Current professional guidelines clearly state that VUS findings should not alter treatment or risk-reducing management and that such patients should instead be followed for possible reclassification [1,2,3]. However, real-world studies have shown inconsistent effects of VUSs on clinical decision-making: some series report no meaningful impact on therapeutic or prophylactic surgery, whereas others suggest that uncertain or non-*BRCA* findings may still influence surgical choices, including contralateral prophylactic mastectomy [14,15,16]. This discrepancy highlights the gap between guideline recommendations and real-world practice and underscores the need to better define the clinicopathological context in which abnormal but non-definitive germline findings are identified.

We recently reported that among patients with abnormal *BRCA1/2* results, pathogenic variants were strongly associated with bilateral mastectomy, whereas surgical decision-making among *BRCA* VUS carriers appeared more heterogeneous and less clearly related to tumor biology [17]. That study, however, focused specifically on *BRCA*-associated surgical patterns. The broader question of whether pathogenic certainty and penetrance level are associated with distinct clinicopathological phenotypes across a wider multigene hereditary breast cancer population remains less well characterized. Accordingly, the aim of this study was to determine whether pathogenic certainty and penetrance level are associated with distinct clinicopathological phenotypes and management patterns in breast cancer patients harboring germline alterations in breast cancer susceptibility genes.

## 2. Methods

### 2.1. Study Design and Setting

This was a retrospective, real-world, observational study conducted in Türkiye. The dataset was assembled from participating oncology, medical genetics, and breast surgery centers involved in the care of patients with breast cancer who underwent germline hereditary cancer testing in routine clinical practice. The participating centers included Göztepe Prof. Dr. Süleyman Yalçın City Hospital, SBÜ Ümraniye Training and Research Hospital, and Istanbul Breast Center.

The structure of the dataset should be interpreted in light of the scope of genetic data captured at each center. *BRCA1/2* pathogenic/likely pathogenic variants and *BRCA1/2* variants of uncertain significance were recorded across the participating centers. In contrast, broader non-*BRCA* homologous recombination repair gene alterations were systematically available from Göztepe Prof. Dr. Süleyman Yalçın City Hospital, where multigene hereditary cancer panel data had been captured in greater detail. Therefore, the treatment-center variable reflected not only the clinical source of the patient but also differences in the scope of genetic data availability. This structure was considered when interpreting center-related effects and when planning multivariable and sensitivity analyses.

The study population comprised 405 patients with breast cancer who had pathogenic/likely pathogenic variants or variants of uncertain significance in breast cancer susceptibility genes. Available demographic, clinicopathological, genetic, and treatment-related variables were extracted retrospectively from institutional records and the study database.

### 2.2. Study Population

Patients were eligible for inclusion if they had a histologically confirmed diagnosis of breast cancer and a germline genetic test result demonstrating either a pathogenic/likely pathogenic (P/LP) variant or a variant of uncertain significance (VUS) in one of the analyzed breast cancer susceptibility genes. Both *BRCA1/2* and non-*BRCA* genes represented in the multigene testing dataset were included. Patients were excluded if they had only benign or likely benign variants, lacked sufficient clinicopathological data for the predefined analyses, or had duplicate records. For analyses focused on tumor phenotype, patients with missing molecular subtype data were excluded from the corresponding model. For VUS-only exploratory analyses, patients harboring any concurrent pathogenic or likely pathogenic variant were excluded so that the VUS cohort reflected genetically uncertain cases only.

### 2.3. Genetic Testing and Variant Classification

All patients had undergone germline testing as part of routine clinical care using peripheral blood-derived DNA and next-generation-sequencing-based hereditary cancer panels in clinical genetic laboratories. The tests were clinically ordered assays rather than custom research panels developed by the investigators. Representative reports in the dataset documented the use of SOPHiA GENETICS hereditary cancer panel solutions, including Hereditary Cancer Solution/Custom Hereditary Cancer Solution panels, with sequencing performed on Illumina platforms, including NextSeq and NovaSeq systems, and bioinformatic analysis performed using Sophia DDM software. The analyzed regions generally included coding exons and adjacent exon–intron boundaries, according to the technical specifications of the reporting laboratory.

The analyzed panels included *BRCA1*, *BRCA2*, and multiple hereditary cancer susceptibility and homologous recombination repair genes, including *ATM*, *BARD1*, *BRIP1*, *CHEK2*, *PALB2*, *RAD51C*, *RAD51D*, and other genes represented in the clinical reports. Variant calling, quality-control filtering, read-depth assessment, and technical interpretation were performed by the reporting laboratories according to their clinical workflows. The investigators did not perform independent raw-sequence variant calling or custom bioinformatic filtering. Because this was a retrospective real-world cohort, the scope of testing was not fully uniform across all reports. *BRCA1/2* pathogenic/likely pathogenic variants and *BRCA1/2* variants of uncertain significance were available across participating centers, whereas broader non-*BRCA* homologous recombination repair gene alterations were systematically available from Göztepe Prof. Dr. Süleyman Yalçın City Hospital. Copy-number variant assessment was also not completely uniform; some reports included *BRCA1/2* CNV analysis, whereas other reports stated that CNV analysis was not performed. Because raw sequencing files were not available, formal sequencing-level batch-effect correction could not be performed. Instead, potential technical heterogeneity related to laboratory workflow, panel scope, and CNV assessment was considered when interpreting the genetic spectrum and was addressed as a limitation of the real-world retrospective design.

Variant classification was based on the final clinical interpretation reported by the medical genetics laboratory and recorded in the study database. The available reports indicated that variant interpretation was performed according to American College of Medical Genetics and Genomics/Association for Molecular Pathology criteria, with use of clinical and population databases such as ClinVar (https://www.ncbi.nlm.nih.gov/clinvar/; accessed on 10 April 2026), OMIM (https://www.omim.org/; accessed on 10 April 2026), dbSNP (https://www.ncbi.nlm.nih.gov/snp/; accessed on 10 April 2026), gnomAD (https://gnomad.broadinstitute.org/; accessed on 10 April 2026), 1000 Genomes (https://www.internationalgenome.org/; accessed on 10 April 2026), and related in silico prediction tools according to the reporting laboratory workflow. Variants were categorized as pathogenic, likely pathogenic, or variant of uncertain significance. Pathogenic and likely pathogenic variants were combined into a single P/LP category for the primary analyses.

Because the present study aimed to examine the relationship between inherited risk architecture and tumor phenotype, genes with P/LP variants were additionally grouped according to their presumed penetrance level. The main genetic exposure variable was therefore defined as a three-level classification: high-penetrance P/LP, moderate/low-penetrance P/LP, and VUS. VUS cases were retained as a distinct category regardless of the host gene, because current clinical interpretation does not equate a VUS finding with confirmed hereditary cancer risk. In patients with more than one reported germline alteration, patients were classified according to the highest-risk confirmed alteration. Accordingly, any high-penetrance P/LP variant overrode lower-risk findings; moderate/low-penetrance P/LP classification was assigned only when no high-penetrance P/LP variant was present; and VUS status was assigned only in the absence of any P/LP alteration.

### 2.4. Definition of Penetrance Groups

For the primary analysis, pathogenic variants were grouped into high-penetrance and moderate/low-penetrance categories in order to preserve statistical stability and maintain clinical interpretability. Genes classified as high penetrance included *BRCA1*, *BRCA2*, *PALB2*, *TP53*, *PTEN*, *CDH1*, and *STK11*. The remaining genes with pathogenic/likely pathogenic alterations in the dataset were classified within the moderate/low-penetrance category. VUS carriers were not reassigned into pathogenic penetrance groups, even when the host gene was considered high penetrance, because the uncertain clinical significance of these variants does not justify management assumptions equivalent to confirmed pathogenic alterations. A prespecified secondary analysis was additionally performed in the non-*BRCA* cohort, comparing non-*BRCA* P/LP carriers with non-*BRCA* VUS carriers to determine whether the observed associations persisted beyond *BRCA*-driven effects.

### 2.5. Data Collection and Clinicopathological Variables

Clinical, pathological, genetic, and treatment-related variables were extracted retrospectively from the study database. The collected variables included age at diagnosis, treatment center, clinical stage at presentation, initial treatment approach, type of breast surgery, final surgical status, family history, histologic subtype, tumor grade, Ki-67 index, estrogen receptor status, progesterone receptor status, HER2 status, molecular subtype, tumor size at presentation, nodal surgery, pathologic response variables when available, prophylactic oophorectomy status, recurrence status, and vital status. Ki-67 values were extracted from routine local pathology reports. No centralized pathology review or central reassessment of Ki-67 immunohistochemistry was performed. Recurrence status was recorded as any documented locoregional or distant recurrence during the available clinical follow-up period. It was not defined at a fixed landmark time point, such as 3 or 5 years. Therefore, recurrence status was treated as a descriptive follow-up variable rather than a primary comparative endpoint. Vital status was recorded according to the most recent available clinical follow-up record. Formal overall survival analysis was not prespecified because survival outcomes were not the primary focus of the study, follow-up duration was not uniform across patients, and death-date information was not consistently complete for all deceased patients. Molecular subtype was analyzed both as a multi-category variable and as a dichotomized phenotype variable. For the primary endpoint analysis, tumors were grouped as luminal (luminal A or luminal B) or non-luminal (HER2-positive or triple-negative). Family history variables were harmonized into clinically analyzable categories for descriptive and regression analyses.

### 2.6. Study Endpoints

The primary endpoint of the study was non-luminal invasive breast cancer phenotype, defined as HER2-positive or triple-negative disease versus luminal A/B disease. The secondary endpoints included age at diagnosis, tumor grade, Ki-67 proliferation index, family history patterns, initial treatment approach, mastectomy versus breast-conserving surgery, final bilateral mastectomy, and prophylactic oophorectomy. A prespecified secondary/sensitivity analysis was conducted in the non-*BRCA* cohort. An exploratory VUS-only analysis was also planned to assess whether host-gene context and variant subtype were associated with clinicopathological features or management patterns.

### 2.7. Statistical Analysis

Descriptive statistics were used to summarize the demographic, clinicopathological, genetic, and treatment-related characteristics of the study population. Continuous variables were reported as median with interquartile range (IQR) and compared using the Kruskal–Wallis test for three-group comparisons or the Mann–Whitney U test for two-group comparisons, as appropriate. Categorical variables were expressed as number and percentage and compared using Pearson’s chi-square test or Fisher’s exact test when expected cell counts were small. The primary analysis evaluated the association between the three-level genetic grouping and the primary endpoint of non-luminal phenotype. For this purpose, binary logistic regression was performed. The main exposure variable was entered as high-penetrance P/LP, moderate/low-penetrance P/LP, and VUS (reference category). The primary multivariable model was adjusted for the clinically relevant covariates age at diagnosis and family history. Treatment center was not included as a routine covariate in the primary genetic phenotype model because center was structurally linked to the scope of genetic data capture: *BRCA1/2* alterations were recorded across participating centers, whereas broader non-*BRCA* HRR-gene alterations were systematically available from Göztepe Prof. Dr. Süleyman Yalçın City Hospital. Therefore, adjustment for center in the primary genetic model could have introduced collinearity or overadjustment. This issue was considered in the interpretation of the results and addressed in sensitivity analyses where appropriate. The primary multivariable model was intentionally parsimonious and adjusted for age at diagnosis and family history, which were selected a priori as clinically relevant baseline variables related to hereditary breast cancer risk and tumor phenotype. Variables that were closely related to the definition of the outcome, including ER status, PR status, HER2 status, molecular subtype, grade, and Ki-67, were not included in the primary model to avoid overadjustment. To evaluate the robustness of the findings, a sensitivity logistic regression model was additionally performed with further adjustment for baseline disease-burden variables, including clinical stage at presentation and clinical/radiological axillary involvement at presentation. Odds ratios (ORs), 95% confidence intervals (CIs), and two-sided *p* values were reported. A prespecified secondary logistic regression analysis was performed in the non-*BRCA* cohort, comparing non-*BRCA* P/LP carriers with non-*BRCA* VUS carriers using the same primary endpoint and adjustment variables. Secondary analyses of management variables were performed descriptively across the three genetic groups. Multivariable modeling for final bilateral mastectomy was planned only if the number of events was sufficient to support a stable model. Exploratory analyses within the VUS-only cohort were performed according to host-gene context and variant subtype. Since recurrence status was not defined at a fixed landmark time point and follow-up duration varied across patients, recurrence was not used as a formal comparative endpoint in the primary analysis. It was summarized descriptively according to available follow-up records. Because recurrence and vital status were recorded during available follow-up rather than according to a uniform landmark time point, these variables were summarized descriptively and were not used as formal time-to-event endpoints. Because these analyses were hypothesis-generating, they were interpreted cautiously, with emphasis placed on effect size direction and descriptive patterns rather than formal causal inference. Missing data were handled using available-case analysis for descriptive statistics and complete-case analysis for multivariable models. The proportion of missing data for key clinicopathological, treatment, and follow-up variables was summarized overall and by pathogenicity/penetrance group and is provided in Table S1. A two-sided *p* value of <0.05 was considered statistically significant. All analyses were performed using IBM SPSS Statistics for Windows, version 26.0 (IBM Corp., Armonk, NY, USA).

### 2.8. Ethical Considerations

The study protocol was reviewed and approved by the Göztepe Prof. Dr. Süleyman Yalçın City Hospital Non-Interventional Clinical Research Ethics Committee (Decision No: 2026/0234; approval date: 9 April 2026). The study was conducted in accordance with the principles of the Declaration of Helsinki. Given the retrospective design and the use of existing clinical and genetic records, the requirement for informed consent was waived by the ethics committee.

## 3. Results

### 3.1. Patient Selection and Analytic Cohorts

A total of 405 breast cancer patients with germline alterations in breast cancer susceptibility genes were included in the study. According to the prespecified three-level classification, 116 patients were categorized as having high-penetrance pathogenic/likely pathogenic (P/LP) variants, 69 patients as having moderate/low-penetrance P/LP variants, and 220 patients as having variants of uncertain significance (VUSs).

For the primary phenotype analysis, patients with ductal carcinoma in situ (DCIS) only and those with missing molecular subtype information were excluded. Accordingly, the analytic cohort for the primary endpoint consisted of 367 patients with evaluable invasive disease. A prespecified secondary analysis was performed in the non-*BRCA* cohort, which comprised 202 patients overall, including 78 non-*BRCA* P/LP carriers and 124 non-*BRCA* VUS carriers; among these, 176 patients had evaluable invasive subtype data. An exploratory analysis was also conducted within the 220-patient VUS cohort. Missing data proportions for key variables are summarized in Table S1. Overall missingness was 15.8% for Ki-67, 16.8% for tumor grade, 7.4% for molecular subtype, 18.3% for initial tumor size, and 9.4% for family history.

### 3.2. Baseline Clinicopathological Characteristics by Pathogenicity/Penetrance Group

Baseline clinicopathological characteristics according to pathogenicity/penetrance group are summarized in Table 1. Patients in the high-penetrance P/LP group were younger at diagnosis than those in the other groups, with a median age of 45 years (IQR, 38–51) compared with 51 years (IQR, 44–58) in the moderate/low-penetrance P/LP group and 51 years (IQR, 45–60) in the VUS group (*p* < 0.001). Clinical stage at presentation was similar across groups (*p* = 0.830). Clinical/radiological axillary involvement at presentation showed a numerical difference, with lower axillary involvement in the moderate/low-penetrance P/LP group, but this did not reach statistical significance (*p* = 0.076).

Initial tumor size did not differ significantly across groups, with median tumor sizes of 23 mm, 22 mm, and 23 mm, respectively (*p* = 0.907). In contrast, proliferative activity differed significantly according to genetic group. Median Ki-67 was highest in the high-penetrance P/LP group (40%, IQR 21–70), compared with 25% (IQR 18–35) in the moderate/low-penetrance P/LP group and 25% (IQR 15–48) in the VUS group (*p* < 0.001). Tumor grade also differed significantly across groups (*p* = 0.012), with grade 3 tumors being more frequent in the high-penetrance P/LP group. Molecular subtype distribution differed significantly among groups (*p* < 0.001). Among patients with non-missing molecular subtype data, triple-negative breast cancer was most frequent in the high-penetrance P/LP group (38/112, 33.9%), compared with 6/61, 9.8% in the moderate/low-penetrance P/LP group and 26/202, 12.9% in the VUS group. Family history also differed across groups. Among patients with known family-history status, a positive family history was present in 74/108 (68.5%) of the high-penetrance P/LP group, 40/56 (71.4%) of the moderate/low-penetrance P/LP group, and 106/203 (52.2%) of the VUS group (*p* = 0.003).

### 3.3. Spectrum of Breast Cancer Susceptibility Genes and Variant Architecture

The spectrum of genes represented in the study cohort is summarized in Table 2A. The most frequently represented genes overall were *BRCA2* (*n* = 124), *BRCA1* (*n* = 79), *CHEK2* (*n* = 43), and *ATM* (*n* = 37). As expected, the high-penetrance P/LP group was dominated by *BRCA2* and *BRCA1*, whereas the moderate/low-penetrance P/LP group consisted mainly of *CHEK2*- and *ATM*-associated cases. Within the VUS group, the most common host genes were *BRCA2*, *BRCA1*, *ATM*, and *CHEK2*.

Variant architecture differed markedly across groups (Table 2B,C). Truncating variants predominated in the high-penetrance P/LP group (91 cases), whereas non-truncating variants were overwhelmingly enriched in the VUS group (191 cases). The moderate/low-penetrance P/LP group showed a more mixed distribution. By specific variant type, missense variants constituted the largest category overall and were especially common among VUS carriers, while frameshift and nonsense variants were largely concentrated in the pathogenic groups. Overall, the distribution of both broad variant architecture and specific variant type differed significantly across the three groups (*p* < 0.001 for both comparisons).

### 3.4. Association of Pathogenicity/Penetrance Group with Non-Luminal Breast Cancer Phenotype

The primary endpoint of the study was non-luminal invasive breast cancer phenotype, defined as HER2-positive or triple-negative disease versus luminal A/B disease. Descriptively, non-luminal tumors were observed in 52/112 (46.4%) of the high-penetrance P/LP group, 18/57 (31.6%) of the moderate/low-penetrance P/LP group, and 75/198 (37.9%) of the VUS group.

In multivariable logistic regression adjusted for age at diagnosis and family history (Table 3), high-penetrance P/LP status was independently associated with higher odds of non-luminal phenotype compared with VUS status (OR 1.79, 95% CI 1.06–3.04; *p* = 0.029). In contrast, moderate/low-penetrance P/LP status did not show a statistically robust association with non-luminal phenotype compared with VUS in this cohort (OR 0.96, 95% CI 0.48–1.91; *p* = 0.911). Because the confidence interval was wide and the moderate/low-penetrance group included biologically heterogeneous genes, this finding should be interpreted cautiously. Neither age at diagnosis nor family history was independently associated with the primary endpoint. In a sensitivity multivariable logistic regression model additionally adjusted for clinical stage at presentation and clinical/radiological axillary involvement at presentation, high-penetrance P/LP status remained associated with non-luminal phenotype compared with VUS (adjusted OR 1.91, 95% CI 1.09–3.33; *p* = 0.023), whereas moderate/low-penetrance P/LP status remained non-significant (adjusted OR 1.02, 95% CI 0.51–2.07; *p* = 0.950) (Table S2).

Because HER2-positive and triple-negative breast cancers represent biologically distinct entities, we performed additional subtype-specific analyses among patients with invasive breast cancer and available molecular subtype data; these analyses are presented in Supplementary Table S3A,B. Triple-negative breast cancer was more frequent in the high-penetrance P/LP group than in the moderate/low-penetrance P/LP and VUS groups (33.9% vs. 10.5% vs. 13.1%, respectively; *p* < 0.001). In contrast, HER2-positive disease was less frequent in the high-penetrance P/LP group than in the moderate/low-penetrance P/LP and VUS groups (12.5% vs. 21.1% vs. 25.3%, respectively; *p* = 0.029).

In subtype-specific multivariable logistic regression analyses adjusted for age at diagnosis and family history, also shown in Supplementary Table S3B, high-penetrance P/LP status was strongly associated with TNBC compared with VUS (adjusted OR 4.68, 95% CI 2.42–9.07; *p* < 0.001). Conversely, high-penetrance P/LP status was associated with lower odds of HER2-positive disease compared with VUS (adjusted OR 0.46, 95% CI 0.23–0.93; *p* = 0.031). Moderate/low-penetrance P/LP status was not significantly associated with either TNBC or HER2-positive disease compared with VUS. These findings suggest that the primary non-luminal association was driven mainly by TNBC enrichment rather than by a uniform increase across all non-luminal subtypes.

### 3.5. Management Patterns Across Pathogenicity/Penetrance Groups

Management patterns are summarized in Table 4. Initial treatment approach did not differ significantly across groups (*p* = 0.577). However, the type of initial breast surgery differed significantly (*p* = 0.003). Initial mastectomy was performed in 70/107 (65.4%) of evaluable patients in the high-penetrance P/LP group, compared with 26/56 (46.4%) in the moderate/low-penetrance P/LP group and 87/191 (45.5%) in the VUS group.

Final bilateral mastectomy differed even more markedly across groups (*p* < 0.001). Bilateral mastectomy was observed in 67/107 (62.6%) of evaluable patients in the high-penetrance P/LP group, compared with 10/57 (17.5%) in the moderate/low-penetrance P/LP group and 19/191 (9.9%) in the VUS group. Similarly, prophylactic oophorectomy was substantially more common in the high-penetrance P/LP group (40/77, 51.9%) than in the moderate/low-penetrance P/LP (3/54, 5.6%) or VUS (9/140, 6.4%) groups (*p* < 0.001).

Because final bilateral mastectomy differed substantially across pathogenicity/penetrance groups, we performed an adjusted logistic regression analysis for this endpoint (Supplementary Table S4A). In the full-cohort model adjusted for age at diagnosis, tumor biology, clinical stage at presentation, and neoadjuvant treatment status, high-penetrance P/LP status remained strongly associated with final bilateral mastectomy compared with VUS (adjusted OR 11.04, 95% CI 5.80–21.02; *p* < 0.001). Moderate/low-penetrance P/LP status showed a numerically higher but statistically non-significant association compared with VUS (adjusted OR 2.04, 95% CI 0.86–4.82; *p* = 0.106). Younger age was also associated with final bilateral mastectomy (adjusted OR per year 0.96, 95% CI 0.93–0.99; *p* = 0.003), whereas tumor biology, clinical stage, and neoadjuvant treatment status were not independently associated with this endpoint in the model.

Treating center was not included in the full-cohort model because the center variable was structurally linked to the scope of genetic data capture, with broader non-*BRCA* HRR-gene alterations systematically available from Göztepe Prof. Dr. Süleyman Yalçın City Hospital. Therefore, we performed a *BRCA*-only sensitivity model including treating center (Table S4B). In this sensitivity analysis, high-penetrance P/LP status remained independently associated with final bilateral mastectomy compared with *BRCA* VUS (adjusted OR 13.31, 95% CI 5.50–32.25; *p* < 0.001).

### 3.6. Secondary Analysis in the Non-BRCA Cohort

A prespecified secondary analysis was conducted in the non-*BRCA* cohort (Table 5A,B). Median age at diagnosis was 52 years (IQR, 45–59) in non-*BRCA* P/LP carriers and 54 years (IQR, 46–61) in non-*BRCA* VUS carriers (*p* = 0.235). Non-luminal phenotype was observed in 19/66 (28.8%) of non-*BRCA* P/LP carriers and 42/110 (38.2%) of non-*BRCA* VUS carriers (*p* = 0.263).

In multivariable analysis adjusted for age and family history, non-*BRCA* P/LP status was not associated with non-luminal phenotype compared with non-*BRCA* VUS (OR 0.83, 95% CI 0.40–1.72; *p* = 0.609). Age showed a borderline association with the endpoint (OR 1.03 per year, 95% CI 1.00–1.07; *p* = 0.058), whereas family history was not associated with non-luminal phenotype.

### 3.7. Exploratory Analysis Among VUS Carriers

Exploratory analyses within the VUS-only cohort are shown in Table 6. Patients with high-penetrance host-gene VUSs were younger at diagnosis than those with moderate/low-penetrance host-gene VUSs (median 50 vs. 53 years; *p* = 0.031). Molecular subtype distribution did not differ significantly between these two VUS subgroups (*p* = 0.799). In contrast, a positive family history was more common among patients with moderate/low-penetrance host-gene VUSs (54/85, 63.5%) than among those with high-penetrance host-gene VUSs (52/118, 44.1%) (*p* = 0.009). Initial surgery type and final bilateral mastectomy rates did not differ significantly between the two VUS host-gene contexts.

## 4. Discussion

In this multicenter retrospective study of 405 breast cancer patients with germline alterations in breast cancer susceptibility genes, we found that pathogenic certainty and penetrance level were associated with meaningful differences in both tumor phenotype and clinical management. Patients carrying high-penetrance pathogenic/likely pathogenic (P/LP) variants were younger at diagnosis, had higher Ki-67 values, and were more likely to have a non-luminal, particularly triple-negative, phenotype than patients with moderate/low-penetrance P/LP variants or VUS. Importantly, the supplementary subtype-specific analyses showed that this non-luminal association was driven primarily by TNBC enrichment rather than by a uniform increase across all non-luminal subtypes. HER2-positive disease did not show the same pattern, supporting the need to interpret HER2-positive and triple-negative tumors separately despite their combined use in the prespecified broad non-luminal endpoint. In multivariable analysis, high-penetrance P/LP status remained independently associated with non-luminal phenotype. By contrast, moderate/low-penetrance P/LP status did not show a statistically robust association in this cohort. However, this null finding should not be interpreted as definitive evidence of absent phenotype specificity, because the moderate/low-penetrance group was smaller, biologically heterogeneous, and underpowered for individual-gene comparisons. Together, these findings support the view that hereditary breast cancer is not a biologically uniform entity and that the clinical meaning of a germline result depends not only on the gene involved, but also on the certainty of pathogenicity and the penetrance context of that gene [9,10,12,13].

Our phenotype findings are consistent with the growing body of literature showing that the pathology of hereditary breast cancer differs substantially by gene. More recent *BRCA*-focused data also suggest that clinicopathologic behavior may vary even within *BRCA1/2*-associated disease according to variant characteristics, further underscoring that hereditary breast cancer biology is not uniform even within traditionally grouped categories [18]. Large-scale studies have demonstrated that *BRCA1*, *BARD1*, *RAD51C*, and *RAD51D* are strongly enriched for triple-negative disease, whereas *ATM* and *CHEK2* are more strongly associated with hormone receptor-positive subtypes; *BRCA2* and *PALB2* occupy an intermediate but clinically important position within this spectrum [8,9,10]. These studies also show that tumors arising in pathogenic-variant carriers are more often higher grade, reinforcing the biologic plausibility of the phenotype gradient observed in our cohort. In this context, the stronger non-luminal signal observed in our high-penetrance group is expected, whereas the weaker and more heterogeneous pattern in the moderate/low-penetrance group likely reflects the fact that these genes do not share a single, uniform breast cancer phenotype.

A particularly informative aspect of our study is the prespecified non-*BRCA* analysis. In the full cohort, the phenotype association appeared to be driven mainly by the high-penetrance P/LP group. However, once *BRCA* carriers were removed, non-*BRCA* P/LP status was no longer associated with non-luminal phenotype. Rather than weakening the study, this finding clarifies the source of the overall signal and argues against extrapolating *BRCA* biology to all hereditary breast cancer genes. Current evidence consistently indicates that moderate-penetrance genes are clinically relevant but far less uniform in their phenotype associations and risk implications than *BRCA1/2* [10,12,13]. Thus, the absence of a strong phenotype signal in the non-*BRCA* analysis is itself clinically meaningful and supports a more nuanced interpretation of multigene panel results.

Another major finding of this study is that VUS behaves as a heterogeneous category rather than a coherent hereditary-risk group. This interpretation is closely aligned with current professional guidance. The ASCO-SSO guideline explicitly states that VUSs should not impact management, and the broader ASCO guidance on multigene panel testing emphasizes that broader panels increase uncertain findings and that the harms of inconclusive results should be mitigated through careful test selection and interpretation [1,2]. Our VUS-only exploratory analysis supports these principles. Although patients with VUSs in high-penetrance host genes were somewhat younger, molecular subtype distribution did not differ significantly by host-gene penetrance context. This suggests that the host gene alone is insufficient to justify phenotype-based assumptions when the variant itself remains unclassified.

At the same time, our management findings show why VUS remains clinically challenging. Although rates of aggressive intervention were substantially lower than in the high-penetrance P/LP group, VUS carriers still represented a group in whom management patterns could not be explained purely on biologic grounds. This is consistent with previous real-world data [19,20]. In our prior BRCA-focused multicenter study, pathogenic *BRCA* status was strongly associated with bilateral mastectomy, whereas among *BRCA* VUS carriers, tumor biology and *BRCA* gene assignment did not clearly explain surgery, and younger age emerged as the main factor associated with treatment choice [17]. Similarly, Murphy et al. reported that in women undergoing multigene panel testing, both non-*BRCA* pathogenic findings and even VUSs were associated with higher rates of contralateral risk-reducing mastectomy [14]. In a more recent safety-net cohort, VUS also remained independently associated with contralateral prophylactic mastectomy, although to a much lesser degree than pathogenic variants [16]. By contrast, Abdel-Razeq et al. found that VUS had no meaningful impact on therapeutic or prophylactic surgical decisions in their regional series [15]. Taken together, these data suggest that the influence of VUS on management is highly context-dependent and may reflect differences in counseling practices, access to genetic expertise, and patient perceptions of uncertainty [14,15,16,17].

The management findings in our full cohort were, however, highly coherent for high-penetrance P/LP carriers. This gene-specific approach to management is also supported by recent contralateral-risk data showing that risk is not uniformly elevated across susceptibility genes, with higher contralateral breast cancer risk reported for *BRCA1*, *BRCA2*, and *CHEK2* carriers, but not clearly for *ATM* carriers, and with more context-dependent findings for *PALB2* [21,22]. These patients underwent mastectomy, bilateral mastectomy, and prophylactic oophorectomy substantially more often than patients in the other two groups. This pattern is consistent with current guideline logic, in which confirmed pathogenic findings in high-penetrance genes may influence local treatment, preventive surgery, and family counseling. It is also compatible with studies showing that testing for actionable non-*BRCA* genes can meaningfully change management recommendations. Vysotskaia et al. showed that testing for genes such as *PALB2*, *ATM*, *CHEK2*, *BRIP1*, *RAD51C*, and *RAD51D* altered eligibility for guideline-based surveillance or preventive strategies in a substantial proportion of carriers [23,24,25]. Likewise, studies of *BRCA*-negative hereditary-risk populations have shown that clinically relevant non-*BRCA* pathogenic variants are identifiable in a meaningful minority of patients, supporting the value of testing beyond *BRCA1/2* alone [26,27,28].

From a clinical perspective, our findings support a risk-stratified interpretation of multigene testing in breast cancer. High-penetrance pathogenic variants appear to define the clearest clinicopathological subgroup, whereas moderate/low-penetrance pathogenic variants and especially VUSs require more cautious interpretation in the broader context of tumor biology, age, and family history. Emerging prospective data further suggest that counseling for carriers of *BRCA1/2*, *ATM*, *CHEK2*, and *PALB2* variants should remain individualized, as many common established exposures do not appear to confer a large uniform increase in breast cancer risk across genes [29,30]. The data therefore reinforce a practical principle already reflected in current guidelines: not all abnormal germline results are biologically or clinically equivalent, and the presence of a variant should not be interpreted apart from its penetrance and classification status [1,2,10,12]. This distinction is becoming increasingly important as broader panel testing is adopted more widely in oncology practice.

This study has several strengths. It includes both *BRCA* and non-*BRCA* alterations, integrates pathogenicity and penetrance into a single analytic framework, and links genetic findings not only to management patterns but also to tumor phenotype. The prespecified non-*BRCA* secondary analysis is particularly valuable because it prevents the full-cohort findings from being interpreted as though they apply uniformly across all hereditary breast cancer genes. Several limitations should also be acknowledged. The retrospective design precluded complete assessment of psychosocial factors, counseling content, and physician-level influences on treatment choice. In addition, several subgroup analyses—particularly within the non-*BRCA* and VUS strata—remained modest in size, and the moderate/low-penetrance group necessarily pooled genes with distinct biologic effects. However, that limitation also reflects current real-world multigene testing practice, in which uncommon but clinically relevant genes are often encountered together rather than in isolation [10,12,23]. Finally, this was a retrospective clinical-report-based study rather than a centralized sequencing study. Although the available reports documented clinical NGS-based hereditary cancer panel testing, panel scope, laboratory workflow, and CNV assessment were not completely uniform across all reports. In addition, the treatment-center variable was partly linked to the scope of genetic data capture, because broader non-*BRCA* HRR-gene alterations were systematically available from Göztepe Prof. Dr. Süleyman Yalçın City Hospital. Therefore, formal sequencing-level batch-effect correction was not possible, and center-adjusted analyses should be interpreted cautiously because of potential collinearity between center and genetic testing scope. The moderate/low-penetrance P/LP group also requires cautious interpretation. This group included a limited number of patients and pooled biologically heterogeneous genes, including genes with different penetrance levels and subtype associations. Therefore, the non-significant association observed for this group may reflect limited statistical power and gene-level heterogeneity rather than a true absence of phenotype specificity. Individual-gene analyses were not performed because event numbers were insufficient for stable estimates.

## 5. Conclusions

Our findings suggest that high-penetrance pathogenic/likely pathogenic germline variants in breast cancer susceptibility genes define the clearest hereditary breast cancer phenotype, characterized by younger age at diagnosis, higher proliferative activity, and a greater likelihood of non-luminal disease. By contrast, moderate/low-penetrance pathogenic variants and especially VUSs do not demonstrate the same degree of phenotype specificity. Together with current guideline recommendations and the broader pathology literature, these data support a framework in which pathogenic certainty and penetrance level remain central to the interpretation of multigene breast cancer testing, while VUS results should remain clearly separated from confirmed hereditary-risk categories in both counseling and management.

## Figures and Tables

**Table 1:** Baseline clinicopathological characteristics by pathogenicity/penetrance group.

Characteristic	High-Penetrance P/LP (*n* = 116)	Moderate/Low-Penetrance P/LP (*n* = 69)	VUS (*n* = 220)	*p* Value
Age at diagnosis, median (IQR)	45 (38–51)	51 (44–58)	51 (45–60)	**<0.001**
Initial tumor size, mm, median (IQR)	23 (16–31)	22 (18–30)	23 (16–33)	0.907
**Clinical stage at presentation**				0.830
Early disease	66/114 (57.9%)	38/61 (62.3%)	116/213 (54.5%)	
Locally advanced disease	41/114 (36.0%)	20/61 (32.8%)	81/213 (38.0%)	
Metastatic disease	7/114 (6.1%)	3/61 (4.9%)	16/213 (7.5%)	
**Clinical/radiological axillary involvement at presentation**				0.076
Absent	47/102 (46.1%)	39/61 (63.9%)	101/202 (50.0%)	
Present	55/102 (53.9%)	22/61 (36.1%)	101/202 (50.0%)	
**Grade**				**0.012**
Grade 1	6/100 (6.0%)	11/58 (19.0%)	26/179 (14.5%)	
Grade 2	36/100 (36.0%)	24/58 (41.4%)	83/179 (46.4%)	
Grade 3	58/100 (58.0%)	23/58 (39.7%)	70/179 (39.1%)	
Ki-67, %, median (IQR)	40 (21–70)	25 (18–35)	25 (15–48)	**<0.001**
**Molecular subtype**				**<0.001**
Luminal A	12/112 (10.7%)	12/61 (19.7%)	45/202 (22.3%)	
Luminal B	48/112 (42.9%)	27/61 (44.3%)	77/202 (38.1%)	
HER2-positive	14/112 (12.5%)	12/61 (19.7%)	50/202 (24.8%)	
Triple-negative	38/112 (33.9%)	6/61 (9.8%)	26/202 (12.9%)	
DCIS	0/112 (0.0%)	4/61 (6.6%)	4/202 (2.0%)	
**Family history**				**0.003**
Present	74/108 (68.5%)	40/56 (71.4%)	106/203 (52.2%)	
Absent	34/108 (31.5%)	16/56 (28.6%)	97/203 (47.8%)	
**Recurrence**				Descriptive only
Present	13/101 (12.9%)	2/63 (3.2%)	19/202 (9.4%)	
Absent	88/101 (87.1%)	61/63 (96.8%)	183/202 (90.6%)	
**Vital status**				Descriptive only
Deceased	8/95 (8.4%)	2/68 (2.9%)	8/200 (4.0%)	
Alive	87/95 (91.6%)	66/68 (97.1%)	192/200 (96.0%)	

Data are presented as median (interquartile range) for continuous variables and number (%) for categorical variables. Percentages were calculated using available cases for each variable; therefore, denominators may vary because of missing data. Continuous variables were compared using the Kruskal–Wallis test, and categorical variables were compared using Pearson’s chi-square test or Fisher’s exact test, as appropriate. Clinical stage at presentation and clinical/radiological axillary involvement were analyzed according to available baseline records. Family history comparisons exclude unknown/missing entries. Bold *p* values indicate statistical significance.

**Table 2:** (**A**). Spectrum of breast cancer susceptibility genes across the study cohort according to pathogenicity/penetrance group. (**B**). Broad variant architecture according to pathogenicity/penetrance group. (**C**). Distribution of specific variant types according to pathogenicity/penetrance group.

**(A)**					
**Gene/Gene Category**	**Overall (*n* = 405)**	**High-Penetrance P/LP**	**Moderate/Low-Penetrance P/LP**	**VUS**	
*BRCA2*	124	60	0	64	
*BRCA1*	79	47	0	32	
*CHEK2*	43	0	28	15	
*ATM*	37	0	15	22	
*PALB2*	13	5	0	8	
*BRIP1*	12	0	2	10	
*BARD1*	11	0	3	8	
*FANCA*	8	0	1	7	
*RAD51D*	8	0	3	5	
*BLM*	8	0	2	6	
*CDH1*	7	1	0	6	
*PTEN*	6	1	0	5	
*ERCC2*	6	0	2	4	
*RAD51C*	5	0	1	4	
Other/rare combinations	38	2	12	24	
**(B)**					
**Variant Architecture**	**Overall (*n* = 399)**	**High-Penetrance P/LP**	**Moderate/Low-Penetrance P/LP**	**VUS**	***p* Value**
Truncating	117	91	21	5	**<0.001**
Non-truncating	242	18	33	191	
Non-coding	40	1	15	24	
**(C)**					
**Variant Type**	**Overall (*n* = 398)**	**High-Penetrance P/LP**	**Moderate/Low-Penetrance P/LP**	**VUS**	***p* Value**
Missense	221	17	26	178	**<0.001**
Frameshift	65	54	8	3	
Nonsense	32	21	10	1	
Splice-site	16	7	8	1	
Indel	15	9	0	6	
Other	49	1	17	31	

(**A**). Data are presented as number of patients. Rare genes and uncommon gene combinations are grouped under “Other/rare combinations” for compact presentation. (**B**). Data are presented as number of patients. Truncating variants include frameshift, nonsense, and canonical splice-site variants where applicable. Non-truncating variants include predominantly missense and in-frame alterations. Non-coding variants include intronic and other non-coding changes. Denominators are lower than the full cohort because variant-architecture annotation was missing in a small number of cases. *p* values were calculated using the chi-square test. (**C**). Data are presented as number of patients. Denominators are lower than the full cohort because detailed variant-type annotation was unavailable in a small number of cases. *p* values were calculated using the chi-square test. Bold *p* values indicate statistical significance.

**Table 3:** Multivariable logistic regression analysis of factors associated with non-luminal breast cancer phenotype in the full cohort.

Variable	Adjusted OR	95% CI	*p* Value
High-penetrance P/LP vs. VUS	1.79	1.06–3.04	**0.029**
Moderate/low-penetrance P/LP vs. VUS	0.96	0.48–1.91	0.911
Age at diagnosis (per year)	1.01	0.99–1.03	0.222
Family history present vs. absent	0.79	0.50–1.26	0.321

The dependent variable was non-luminal phenotype, defined as HER2-positive or triple-negative disease versus luminal A/B disease. The reference category for genetic grouping was VUS. Odds ratios (ORs) and 95% confidence intervals (CIs) were obtained from binary logistic regression analysis. The model was adjusted for age at diagnosis and family history. Complete-case analysis was used. Bold *p* values indicate statistical significance.

**Table 4:** Management patterns across pathogenicity/penetrance groups.

Management Variable	High-Penetrance P/LP	Moderate/Low-Penetrance P/LP	VUS	*p* Value
Initial approach				0.577
Upfront surgery	50/115 (43.5%)	35/62 (56.5%)	103/212 (48.6%)	
Neoadjuvant treatment	57/115 (49.6%)	24/62 (38.7%)	94/212 (44.3%)	
Systemic treatment	8/115 (7.0%)	3/62 (4.8%)	15/212 (7.1%)	
Initial surgery				**0.003**
Mastectomy	70/107 (65.4%)	26/56 (46.4%)	87/191 (45.5%)	
Breast-conserving surgery	37/107 (34.6%)	30/56 (53.6%)	104/191 (54.5%)	
Final surgery				**<0.001**
Bilateral mastectomy	67/107 (62.6%)	10/57 (17.5%)	19/191 (9.9%)	
Non-bilateral surgery	40/107 (37.4%)	47/57 (82.5%)	172/191 (90.1%)	
Prophylactic oophorectomy				**<0.001**
Performed	40/77 (51.9%)	3/54 (5.6%)	9/140 (6.4%)	
Not performed	37/77 (48.1%)	51/54 (94.4%)	131/140 (93.6%)	

Data are presented as number (%) based on available cases for each variable. Categorical comparisons were performed using Pearson’s chi-square test or Fisher’s exact test, as appropriate. Denominators vary because of missing data. Bold *p* values indicate statistical significance.

**Table 5:** (**A**). Descriptive clinicopathological comparison between non-*BRCA* pathogenic/likely pathogenic and non-*BRCA* VUS carriers. (**B**). Multivariable logistic regression analysis of non-luminal phenotype in the non-*BRCA* cohort.

**(A)**			
**Variable**	**Non-*BRCA* P/LP**	**Non-*BRCA* VUS**	***p* Value**
Age at diagnosis, median (IQR)	52 (45–59)	54 (46–61)	0.235
Luminal phenotype	47/66 (71.2%)	68/110 (61.8%)	0.263
Non-luminal phenotype	19/66 (28.8%)	42/110 (38.2%)	
Family history present	45/63 (71.4%)	69/108 (63.9%)	0.400
Family history absent	18/63 (28.6%)	39/108 (36.1%)	
**(B)**			
**Variable**	**Adjusted OR**	**95% CI**	***p*** **Value**
non-*BRCA* P/LP vs. non-*BRCA* VUS	0.83	0.40–1.72	0.609
Age at diagnosis (per year)	1.03	1.00–1.07	0.058
Family history present vs. absent	0.74	0.36–1.55	0.430

(**A**). Data are presented as median (interquartile range) for continuous variables and number (%) for categorical variables. Family history comparisons exclude unknown/missing entries. Continuous variables were compared using the Mann–Whitney U test, and categorical variables were compared using Pearson’s chi-square test or Fisher’s exact test, as appropriate. (**B**). The dependent variable was non-luminal phenotype, defined as HER2-positive or triple-negative disease versus luminal A/B disease. The reference category was non-*BRCA* VUS. Odds ratios (ORs) and 95% confidence intervals (CIs) were obtained from binary logistic regression analysis adjusted for age at diagnosis and family history. Complete-case analysis was used.

**Table 6:** Exploratory analysis of clinicopathological characteristics among VUS carriers according to host-gene penetrance context.

Variable	High-Penetrance Host-Gene VUS	Moderate/Low-Penetrance Host-Gene VUS	*p* Value
Age at diagnosis, median (IQR)	50 (42–60)	53 (47–60)	**0.031**
Luminal A	24/110 (21.8%)	21/92 (22.8%)	0.799
Luminal B	44/110 (40.0%)	33/92 (35.9%)	
HER2-positive	27/110 (24.5%)	23/92 (25.0%)	
Triple-negative	14/110 (12.7%)	12/92 (13.0%)	
DCIS	1/110 (0.9%)	3/92 (3.3%)	
Family history present	52/118 (44.1%)	54/85 (63.5%)	**0.009**
Family history absent	66/118 (55.9%)	31/85 (36.5%)	
Initial mastectomy	50/106 (47.2%)	37/85 (43.5%)	0.722
Initial breast-conserving surgery	56/106 (52.8%)	48/85 (56.5%)	
Final bilateral mastectomy	13/106 (12.3%)	6/85 (7.1%)	0.341
Final non-bilateral surgery	93/106 (87.7%)	79/85 (92.9%)	

Data are presented as median (interquartile range) for continuous variables and number (%) for categorical variables. Family history comparisons exclude unknown/missing entries. Continuous variables were compared using the Mann–Whitney *U* test, and categorical variables were compared using Pearson’s chi-square test or Fisher’s exact test, as appropriate. These analyses were exploratory and should be interpreted cautiously. Bold *p* values indicate statistical significance.

## Data Availability

The datasets used and/or analyzed during the current study are available from the corresponding author.

## Supplementary Materials

The following supporting information can be downloaded at: https://www.mdpi.com/article/10.3390/cancers18101499/s1, Table S1: Missing data according to pathogenicity/penetrance group; Table S2: Sensitivity multivariable logistic regression analysis of factors associated with non-luminal breast cancer phenotype; Table S3A: Separate distribution of triple-negative and HER2-positive breast cancer according to pathogenicity/penetrance group; Table S3B: Subtype-specific multivariable logistic regression analyses for triple-negative and HER2-positive breast cancer; Table S4A: Multivariable logistic regression analysis of factors associated with final bilateral mastectomy in the full cohort; Table S4B: BRCA-only sensitivity model including treating center for final bilateral mastectomy.
