# Supplementary material for: Risk Tier, Variant Certainty, and Real-World Care Patterns in Breast Cancer Patients with Germline Alterations in Breast Cancer Susceptibility Genes
Source: Cancers (Basel). 2026 May 7;18(10):1499. doi: 10.3390/cancers18101499 (PMC13204001; doi:10.3390/cancers18101499)
Supplement: Supplementary file 1 [file cancers-18-01499-s001.zip › cancers-4284624-supplementary.pdf]

**Supplementary Table S1**. Missing data according to pathogenicity/penetrance group.

| Variable | Overall (n=405) | High-penetrance P/LP (n=116) | Moderate/low-penetrance P/LP (n=69) | VUS (n=220) |
|---|---|---|---|---|
| **Age at diagnosis** | 0/405 (0.0%) | 0/116 (0.0%) | 0/69 (0.0%) | 0/220 (0.0%) |
| **Initial tumor size at presentation** | 74/405 (18.3%) | 20/116 (17.2%) | 16/69 (23.2%) | 38/220 (17.3%) |
| **Clinical stage at presentation** | 17/405 (4.2%) | 2/116 (1.7%) | 8/69 (11.6%) | 7/220 (3.2%) |
| **Clinical/radiological axillary involvement at presentation** | 40/405 (9.9%) | 14/116 (12.1%) | 8/69 (11.6%) | 18/220 (8.2%) |
| **Tumor grade** | 68/405 (16.8%) | 16/116 (13.8%) | 11/69 (15.9%) | 41/220 (18.6%) |
| **Ki-67 index** | 64/405 (15.8%) | 14/116 (12.1%) | 11/69 (15.9%) | 39/220 (17.7%) |
| **Molecular subtype** | 30/405 (7.4%) | 4/116 (3.4%) | 8/69 (11.6%) | 18/220 (8.2%) |
| **Family history** | 38/405 (9.4%) | 8/116 (6.9%) | 13/69 (18.8%) | 17/220 (7.7%) |
| **Initial treatment approach** | 16/405 (4.0%) | 1/116 (0.9%) | 7/69 (10.1%) | 8/220 (3.6%) |
| **Initial surgery type** | 51/405 (12.6%) | 9/116 (7.8%) | 13/69 (18.8%) | 29/220 (13.2%) |
| **Final surgery status** | 50/405 (12.3%) | 9/116 (7.8%) | 12/69 (17.4%) | 29/220 (13.2%) |
| **Prophylactic oophorectomy status** | 134/405 (33.1%) | 39/116 (33.6%) | 15/69 (21.7%) | 80/220 (36.4%) |
| **Recurrence status** | 39/405 (9.6%) | 15/116 (12.9%) | 6/69 (8.7%) | 18/220 (8.2%) |
| **Vital status** | 42/405 (10.4%) | 21/116 (18.1%) | 1/69 (1.4%) | 20/220 (9.1%) |

**Footnote:** Data are presented as number of missing values/total number of patients (%). For family history, unknown entries were treated as missing for analytic purposes. Missingness was assessed according to the variables used in the main descriptive, regression, management, and follow-up summaries.

**Supplementary Table S2**. Sensitivity multivariable logistic regression analysis of factors associated with non-luminal breast cancer phenotype.

| Variable | Adjusted OR | 95% CI | p value |
|---|---|---|---|
| **High-penetrance P/LP vs. VUS** | 1.91 | 1.09–3.33 | **0.023** |
| **Moderate/low-penetrance P/LP vs. VUS** | 1.02 | 0.51–2.07 | 0.950 |
| **Age at diagnosis, per year** | 1.01 | 0.99–1.03 | 0.177 |
| **Family history present vs. absent** | 0.74 | 0.45–1.21 | 0.234 |
| **Locally advanced vs. early clinical stage** | 2.34 | 1.01–5.40 | **0.047** |
| **Metastatic vs. early clinical stage** | 0.36 | 0.04–3.21 | 0.359 |
| **Clinical/radiological axillary involvement present vs. absent** | 0.50 | 0.22–1.14 | 0.098 |

**Footnote:** The dependent variable was non-luminal phenotype, defined as HER2-positive or triple-negative invasive breast cancer versus luminal A/B disease. The reference category for genetic grouping was VUS. The model was adjusted for age at diagnosis, family history, clinical stage at presentation, and clinical/radiological axillary involvement at presentation. Complete-case analysis was used.

**Supplementary Table S3A.** Separate distribution of triple-negative and HER2-positive breast cancer according to pathogenicity/penetrance group.

| Subtype-specific phenotype | High-penetrance P/LP | Moderate/low-penetrance P/LP | VUS | p value |
|---|---|---|---|---|
| **Triple-negative breast cancer** | | | | **<0.001** |
| **Present** | 38/112 (33.9%) | 6/57 (10.5%) | 26/198 (13.1%) | |
| **Absent** | 74/112 (66.1%) | 51/57 (89.5%) | 172/198 (86.9%) | |
| **HER2-positive breast cancer** | | | | **0.029** |
| **Present** | 14/112 (12.5%) | 12/57 (21.1%) | 50/198 (25.3%) | |
| **Absent** | 98/112 (87.5%) | 45/57 (78.9%) | 148/198 (74.7%) | |

**Footnote:** Analyses were restricted to patients with invasive breast cancer and available molecular subtype data. DCIS and patients with missing molecular subtype were excluded. TNBC was compared with all other invasive subtypes, and HER2-positive disease was compared with all other invasive subtypes. Categorical comparisons were performed using Pearson's chi-square test. Bold p values indicate statistical significance.

**Supplementary Table S3B.** Subtype-specific multivariable logistic regression analyses for triple-negative and HER2-positive breast cancer.

| Outcome | Variable | Adjusted OR | 95% CI | p value |
|---|---|---|---|---|
| **Triple-negative breast cancer vs. non-TNBC invasive disease** | High-penetrance P/LP vs. VUS | 4.68 | 2.42–9.07 | **<0.001** |
| | Moderate/low-penetrance P/LP vs. VUS | 1.02 | 0.36–2.88 | 0.977 |
| | Age at diagnosis, per year | 1.01 | 0.98–1.03 | 0.643 |
| | Family history present vs. absent | 0.46 | 0.25–0.84 | **0.011** |
| **HER2-positive breast cancer vs. non-HER2-positive invasive disease** | High-penetrance P/LP vs. VUS | 0.46 | 0.23–0.93 | **0.031** |
| | Moderate/low-penetrance P/LP vs. VUS | 0.88 | 0.41–1.91 | 0.756 |
| | Age at diagnosis, per year | 1.01 | 0.99–1.04 | 0.262 |
| | Family history present vs. absent | 1.36 | 0.77–2.39 | 0.290 |

**Footnote:** Binary logistic regression was performed using complete-case analysis among patients with invasive breast cancer and available molecular subtype data. The TNBC model compared triple-negative breast cancer with all other invasive subtypes. The HER2-positive model compared HER2-positive breast cancer with all other invasive subtypes. The reference category for genetic grouping was VUS. Models were adjusted for age at diagnosis and family history. OR, odds ratio; CI, confidence interval; P/LP, pathogenic/likely pathogenic; VUS, variant of uncertain significance; TNBC, triple-negative breast cancer. Bold p values indicate statistical significance.

**Supplementary Table S4A.** Multivariable logistic regression analysis of factors associated with final bilateral mastectomy in the full cohort.

| Variable | Adjusted OR | 95% CI | p value |
|---|---|---|---|
| **High-penetrance P/LP vs. VUS** | 11.04 | 5.80–21.02 | **<0.001** |
| **Moderate/low-penetrance P/LP vs. VUS** | 2.04 | 0.86–4.82 | 0.106 |

| | | | |
|---|---|---|---|
| **Age at diagnosis, per year** | 0.96 | 0.93–0.99 | **0.003** |
| **Non-luminal vs. luminal tumor biology** | 1.38 | 0.75–2.53 | 0.297 |
| **Advanced/metastatic vs. early clinical stage** | 0.97 | 0.48–1.96 | 0.925 |
| **Neoadjuvant treatment received vs. not received** | 1.58 | 0.77–3.26 | 0.215 |

**Footnote:** The dependent variable was final bilateral mastectomy versus non-bilateral surgery. Binary logistic regression was performed using complete-case analysis. The model included genetic group, age at diagnosis, tumor biology, clinical stage at presentation, and neoadjuvant treatment status. Tumor biology was entered as luminal versus non-luminal. Clinical stage was dichotomized as early versus advanced/metastatic to preserve model stability. The reference category for genetic grouping was VUS. OR, odds ratio; CI, confidence interval; P/LP, pathogenic/likely pathogenic; VUS, variant of uncertain significance. Bold p values indicate statistical significance.

**Supplementary Table S4B.** BRCA-only sensitivity model including treating center for final bilateral mastectomy.

| Variable | Adjusted OR | 95% CI | p value |
|---|---|---|---|
| **High-penetrance P/LP vs. BRCA VUS** | 13.31 | 5.50–32.25 | **<0.001** |
| **Age at diagnosis, per year** | 0.98 | 0.94–1.02 | 0.231 |
| **Non-luminal vs. luminal tumor biology** | 1.52 | 0.69–3.39 | 0.301 |
| **Advanced/metastatic vs. early clinical stage** | 1.99 | 0.75–5.31 | 0.167 |
| **Neoadjuvant treatment received vs. not received** | 1.02 | 0.40–2.59 | 0.973 |
| **Center 2 vs. Center 1** | 5.99 | 2.02–17.70 | **0.001** |
| **Center 3 vs. Center 1** | 0.22 | 0.05–0.93 | **0.040** |

**Footnote:** This sensitivity model was restricted to BRCA-associated cases because treating-center information was available for the BRCA subset, whereas non-*BRCA*/HRR data were structurally linked to the data-source structure. The dependent variable was final bilateral mastectomy versus non-bilateral surgery. The model was adjusted for age at diagnosis, tumor biology, clinical stage, neoadjuvant treatment status, and treating center. The reference genetic category was BRCA VUS. Center 1 represents Göztepe Prof. Dr. Süleyman Yalçın City Hospital, Center 2 Istanbul Breast Center and Center 3 SBÜ Ümraniye Training and Research Hospital.
